# Toward diagnostic preparedness: detection of highly pathogenic avian influenza A(H5N1) in contrived nasal swab specimens using rapid antigen and point-of-care molecular tests

**DOI:** 10.1128/jcm.00548-25

**Published:** 2025-08-19

**Authors:** Leda Bassit, Gregory L. Damhorst, Heather B. Bowers, Courtney Sabino, Julie Sullivan, Emily B. Kennedy, Jacob Khouri, Pamela Miller, Eric Lai, Raymond F. Schinazi, Wilbur A. Lam, Nira R. Pollock, Anuradha Rao

**Affiliations:** 1 Center for ViroScience and Cure, Laboratory of Biochemical Pharmacology, Department of Pediatrics, Emory University School of Medicine 12239, Atlanta, Georgia, USA; 2 The Atlanta Center for Microsystems-Engineered Point-of-Care TechnologiesAtlanta, Georgia, USA; 3 Division of Infectious Diseases, Emory University School of Medicine 12239, Atlanta, Georgia, USA; 4 Department of Pediatrics, Emory University School of Medicine 12239, Atlanta, Georgia, USA; 5 OOMVELTLakewood, Ohio, USA; 6 Breton Highlands Consulting 252918 Sand Springs, Oklahoma, USA; 7 Echo ConsultingExcelsior, Minnesota, USA; 8 PharmaDx, LLCSan Diego, California, USA; 9 Aflac Cancer & Blood Disorders Center at Children's Healthcare of Atlanta 584996 Atlanta, Georgia, USA; 10 Wallace H. Coulter Department of Biomedical Engineering, Georgia Institute of Technology 189277 Atlanta, Georgia, USA; 11 National Institute of Biomedical Imaging and Bioengineering, National Institutes of Health 2511, Bethesda, Maryland, USA; Wadsworth Center - NYSDOH, Albany, New York, USA

**Keywords:** influenza A, POC, OTC, bird flu, lateral flow assays

## Abstract

Highly pathogenic avian influenza (HPAI) A(H5N1) clade 2.3.4.4b was first detected in birds in 2021 in the United States (U.S.). An ongoing outbreak in dairy cattle began in early 2024 involving genotype B3.13. At least 70 U.S. cases have been identified in humans with exposure to infected cattle, poultry, and wild birds. No human-to-human transmission has yet been documented. However, for diagnostic preparedness, we evaluated the ability of currently available influenza tests to detect 2024 U.S. H5N1 genotypes, B3.13 and D1.1, predominant in wild birds and poultry. Contrived nasal swab samples were prepared using live or inactivated virus and used to test 12 rapid antigen tests (lateral flow assays, [LFA]), including 10 commercially available influenza A and two H5-specific LFAs. Five point-of-care (POC) molecular assays were also tested. An inclusivity testing protocol utilizing a predetermined dilution series was used to evaluate each assay, enabling a head-to-head comparison of performances. All LFAs and POC molecular tests were able to detect bovine 2024 H5N1 genotype B3.13. Sensitivity for the POC molecular tests (heat-inactivated B3.13) ranged from 1.55 to 7.75 TCID_50_/swab. For 11/12 LFAs, 10 commercial influenza A, and an RUO H5 assay, the sensitivity (live B3.13) ranged from 78 to 1550 TCID_50_/swab. The 12th LFA detected 77,500 TCID_50_/mL transport media. Testing of four LFAs confirmed inclusivity for genotype D1.1. Available influenza tests can detect 2024 U.S. H5N1 strains in contrived samples with a wide range of analytical sensitivity. In the event of human-to-human transmission, clinical performance and optimal sample types would need to be established.

**IMPORTANCE:**

To date, at least 70 human cases of highly pathogenic avian influenza A (HPAI) H5N1 have been reported. Human-to-human transmission has not yet been reported, but there is ongoing HPAI H5N1 spread within animal and bird populations. As part of pandemic preparedness, it is critical to know whether available tests capable of detecting seasonal influenza A can also detect HPAI H5N1. Our paper describes results from the first study to assess and compare the ability of 10 commercially available influenza A lateral flow assays (LFAs), two LFAs specific for H5 influenza, and five POC molecular tests from three manufacturers to detect the H5N1 virus circulating in U.S. cattle (genotype B3.13). We found that all tests detect HPAI H5N1 in contrived nasal swab specimens, with varying degrees of analytical sensitivity. We also tested a subset of LFAs (three 510k-cleared COVID/flu multiplex tests and an RUO H5-specific LFA) to confirm that they were able to detect a D1.1 genotype (the predominant genotype in wild birds and poultry) strain isolated from a human case of H5N1 in Washington State. Given the persistent circulation of HPAI H5N1 cases, it is critical to know that available tests can analytically detect H5N1. Clinical performance and optimal sample types should be evaluated if human-to-human transmission is observed.

## INTRODUCTION

Highly pathogenic avian influenza (HPAI) A(H5N1) has been detected sporadically in humans since 1997 (1). H5N1 clade 2.3.4.4b, which has been present in birds in the United States (U.S.) since 2021, was identified as the cause of a non-specific syndrome among dairy cattle in the U.S. in early 2024 (2). Since then, at least 70 confirmed human cases have been identified in the United States, primarily among individuals with exposure to cattle, poultry, or wild birds, including a severe case leading to hospitalization and death (3–5). Although the public health risk is currently considered to be low, these patterns have prompted preparedness efforts acknowledging the potential for spontaneous mutations or reassortment with seasonal influenza A leading to widespread human-to-human transmission of HPAI H5N1.

The critical role of diagnostics in pandemic preparedness was a major lesson of the severe acute respiratory syndrome coronavirus 2 (SARS-CoV-2) pandemic. The public health response facilitated an acceleration in diagnostic test innovation that shifted testing paradigms from limited centralized testing resources at the onset of the pandemic to widespread point-of-care (POC) and over-the-counter (OTC) diagnostics (6, 7). This momentum has since also fueled advances in influenza diagnostics, as the first commercial OTC self-tests for influenza A and B have been cleared by the United States Food and Drug Administration (FDA) (8) in the form of multiplexed rapid antigen lateral flow assays (LFAs) also capable of detecting SARS-CoV-2. Currently, five home-use multiplex SARS-CoV-2/influenza LFAs have FDA Emergency Use Authorization (EUA), and three have received marketing authorization through traditional FDA 510k pathways (9–11). POC molecular tests for influenza have been available since 2015 and generally offer superior sensitivity for the detection of influenza virus infection compared to rapid antigen tests (12, 13).

Diagnostic preparedness for the possibility of widespread H5N1 transmission among humans requires verification that the current strains circulating in cattle and birds are detected by available commercial tests. Many existing OTC/POC assays are expected to detect earlier H5N1 strains on the basis of *in silico* or direct inclusivity testing reported in instructions for use (IFU); however, these data do not report specifically on clade 2.3.4.4b (14). As an early indicator of the inclusivity of existing OTC and POC tests for the clade currently circulating in the U.S. cattle and poultry and to enable a head-to-head comparison of available tests’ performances for HPAI detection, we tested commercially available assays with contrived specimens prepared with live or inactivated representative HPAI H5N1 strains [A/bovine/Ohio/B24OSU-439/2024 (H5N1), genotype B3.13, and A/Washington/240/2024, genotype D1.1]. While both strains are within H5N1 clade 2.3.4.4b, genotype B3.13 is the primary genotype associated with outbreaks in dairy cattle in the U.S., and genotype D1.1 is the predominant genotype in migratory birds and poultry in North America. The B3.13 strain evaluated was isolated from a dairy cow on 5 April 2024 in Ohio, U.S., and was the first strain available for use from Biodefense and Emerging Infections Research Resources Repository (BEI Resources). The D1.1 strain was isolated from a sample from a human case related to bird exposure in Washington State, and testing whether this strain could be detected by LFAs was important given the ongoing risk of transmission from birds to humans.

## MATERIALS AND METHODS

### Influenza viruses

Tissue culture-propagated HPAI A virus/bovine/Ohio/B24OSU-439/2024 (H5N1) (GISAID ID- EPI_ISL_19178083) was obtained from BEI Resources (NR-59872, lot 70070368). This virus was propagated in Madin-Darby canine kidney (MDCK) cells and provided at a concentration of 3.1E6 TCID_50_/mL determined by the cytopathic effect in MDCK cells by the manufacturer. This H5N1 clade 2.3.4.4b, genotype B3.13 strain is denoted as “2024 HPAI H5N1” in this article. Another H5N1 clade 2.3.4.4b strain with genotype D1.1(A/Washington/240/2024) (GISAID ID- EPI_ISL_19531299) was provided by the Centers for Disease Control and Prevention (CDC) at a concentration of 2.37E10 EID50/mL. This strain was isolated from a sample from a human case in Washington State and is denoted in this article as “2024 HPAI H5N1 D1.1.” A 2009 H5N1 strain, A/mallard/Wisconsin/2576/2009 (H5N1), was obtained from BEI Resources (NR-31131, lot 61788259).

Procedures involving live 2009 H5N1 and 2024 HPAI H5N1 strains (live virus being optimal for testing of LFAs detecting protein antigens, as heat inactivation may denature proteins), including testing of lateral flow assays (LFAs), were performed in Emory University’s biosafety level (BSL)-3 laboratory using personal protective equipment according to standards set by the CDC and the Emory University Environmental Health and Safety Office. For select LFAs, testing was also performed with an inactivated gamma-irradiated (GIV) version of the BEI strain (NR- 59886, lot 70070836) to compare detection of live versus irradiated virus.

Additional testing for select LFAs (Healgen Rapid Check COVID-19/Flu A&B Antigen Test, ACON Laboratories-Flowflex Plus COVID-19 and Flu A/B Home Test, and LFA B) was carried out using the following live strains: A/California/04/2009 (H1N1), A/Ohio/09/2015 (H1N1), A/Tasmania/503/2020 (H3N2), and A/Indiana/08/2011 (H3N2).

Because it was impractical to bring POC molecular platforms into the BSL3 facility, heat-inactivated 2024 HPAI H5N1 was prepared for testing of these platforms by heating live virus at 60°C for 30 min. Inactivation was confirmed by lack of virus propagation and no evidence of cell death (by crystal violet staining) in MDCK cells infected with 100 µL of 10 serial fivefold dilutions starting at a virus concentration of 3.1E4 TCID_50_/mL.

For all strains, the inclusivity protocol described below in detail for LFAs and molecular assays was followed.

### Negative nasal swab matrix preparation

Pooled negative nasal swab matrix (PNSM) was used as diluent for all influenza viruses tested. Details are provided in the supplemental methods.

### Contrived specimen preparation

2024 HPAI H5N1 and 2024 HPAI H5N1 D1.1 stocks were thawed in a BSL-3 facility and aliquoted in 50 µL aliquots, which were then frozen at −80°C. For LFA testing, aliquots (live virus or GIV) were thawed and diluted to the intended final concentrations in PNSM. Then, using a precision pipette, 50 µL of each dilution was pipetted onto the respective assay swab to simulate the collection of a nasal sample, and the assay IFU was followed to test that swab. For molecular assays, 50 µL of heat-inactivated 2024 HPAI H5N1 diluted in PNSM was first pipetted onto the respective assay swab. For the Abbott ID NOW assays, the swab with added sample was tested directly according to the IFU. For assays requiring an intermediate elution of the swab in transport medium, swabs were eluted in 3 mL of UTM Universal Transport Medium (3C047N, Copan Diagnostics). These contrived UTM specimens were aliquoted and frozen to facilitate workflow. The UTM specimen aliquots were thawed just before testing. All viral concentrations tested were calculated from the TCID_50_/mL provided by the stock manufacturer.

### LFA inclusivity testing

Inclusivity studies (studies designed to answer the question of whether a test recognizes a different strain of the intended target) were performed according to the routine regulatory procedure by first testing contrived samples with the live virus at 10-fold dilutions (for 2024 HPAI H5N1: 310,000; 31,000; 3,100; and 310 TCID_50_/mL [corresponding to 15,500; 1,550; 155; and 15.5 TCID_50_ per swab]), each in triplicate. The lowest dilution for which all three replicates generated a positive result was then used to create three additional twofold serial dilutions, each of which was tested in triplicate. The lowest dilution detected in all three replicates was defined as the lowest concentration detectable through inclusivity testing. The same approach was used for testing select LFAs with GIV. The 10 commercially available influenza A LFA assays tested in this manner included eight for which the manufacturer granted permission to disclose the device name in the publication and two for which the manufacturer requested that the name be redacted. Two H5-specific LFAs (Healgen H5 RUO and Arbor Vita AV Avantage A/H5N1) were also evaluated using this protocol (see the supplemental methods for details). All of the LFAs were visually read, except for one (Quidel Sofia Flu + SARS Antigen FIA), a POC LFA which requires a reader.

### Molecular assay inclusivity testing

The molecular assays tested (heat-inactivated virus) included the Cepheid Xpert Xpress CoV-2/Flu/RSV *plus* test (EUA201505) with the Cepheid GeneXpert systems (a GeneXpert XVI platform, which is equivalent in this context to the GeneXpert Xpress authorized for waived settings, was used). (EUA cartridge reagents and software are identical to the 510k-approved product with FDA parent document number K231481.) Additional assays evaluated included the Roche cobas Influenza A/B & RSV (K213822) and Roche cobas SARS-CoV-2 & Influenza A/B (K223591) with the cobas Liat System and the Abbott ID NOW Influenza A&B 2 (K191534) and Abbott ID NOW COVID-19 2.0 with Influenza A&B 2 sequential workflow (in which a single patient swab is first tested for SARS-CoV-2 [K221925] and then reflexed to Flu A/B testing [K191534]). For each assay, PNSM contrived with heat-inactivated 2024 HPAI H5N1 virus at 310, 31, and 3.1 TCID_50_/mL (15.5, 1.55, and 0.155 TCID_50_ per swab) was introduced as a simulated nasal swab and processed according to the assay IFU, with intermediate elution in 3 mL of UTM where indicated.

### CDC assay

**Fig 1:**
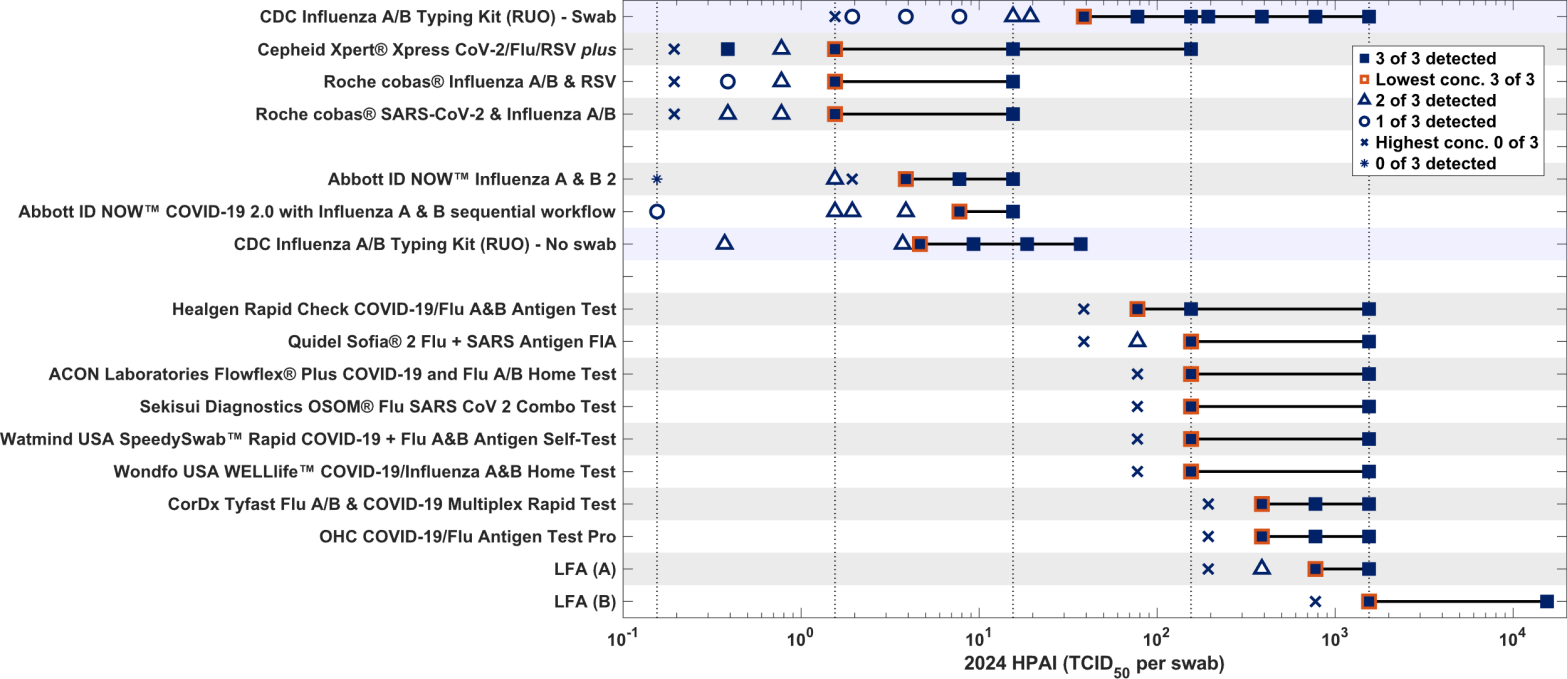
Performance of five POC molecular tests and 10 LFAs for detection of 2024 HPAI H5N1 in contrived nasal swab specimens. The CDC Influenza A/B typing kit (CDC Human Influenza Virus Real-time RT-PCR Diagnostic Panel, Influenza A/B Typing Kit) was performed in two different ways to facilitate direct comparison to POC molecular assays with different formats. For direct comparison to POC molecular assays utilizing swab samples eluted in UTM, 120 μL of the UTM sample used for the POC molecular assays was tested with the CDC assay (“swab”). For direct comparison to assays not requiring UTM, 120 ul of the PNSM sample used for the POC molecular assays was tested with the CDC assay (“no swab”). The first (most concentrated) sample to yield 0 of 3 positive results is indicated by ‘X’. For Abbott ID NOW Influenza A & B 2, the next concentration with 0 of 3 positive results is indicated by “*”. Lower dilutions for which 0 of 3 replicates were positive are indicated in Tables 1 to 3. Two LFAs (LFA A and B) were anonymized at the request of the companies. UTM, Universal Transport Media; LFA, Lateral Flow Assay. Data for H5 specific LFAs were not included in this figure.

The CDC Influenza A/B Typing Kit (IVD Kit, K190302, CDC Human Influenza Virus Real-time RT-PCR Diagnostic Panel) was employed as a research-use only (RUO) application according to the IFU. The CDC assay was performed in two different ways to facilitate direct comparison to POC molecular assays with different testing formats. For direct comparison to POC molecular assays utilizing swab samples eluted in UTM (Cepheid Xpert Xpress CoV-2/Flu/RSV *plus* [350 μL] and Roche cobas Influenza A/B & RSV and Roche cobas SARS-CoV-2 & Influenza A/B assays [200 μL each]), 120 μL of the UTM sample was tested with the CDC assay (“Swab” in Fig. 1). For a direct comparison to the Abbott ID NOW Influenza A & B 2 and Abbott ID NOW COVID-19 2.0 with Influenza A & B 2 sequential workflow systems (which utilize 50 μL sample in PNSM directly pipetted to a swab), 120 μL of the PNSM sample was tested with the CDC assay (“No swab” in Fig. 1). RNA was isolated using the Qiagen EZ1 DSP Viral Kit (utilizing 280 µL of AVL buffer and 120 µL of elution buffer). For RT-qPCR, the Invitrogen SuperScript III Platinum One-Step qRT-PCR System was used, utilizing 5 µL RNA and 20 µL PCR master mix per reaction and an Applied Biosystems 7500 Fast instrument for amplification.

### Digital droplet PCR (ddPCR)

For determination of genomic equivalents (GE)/mL by ddPCR, we used the InfA Primers/Probe from the IVD CDC H5 Subtyping Kit. The One-Step RT-ddPCR Advanced Kit for Probes (BioRAD) was used according to the manufacturer’s instructions, with 5 µL of RNA and 17 µL of master mix per reaction. An aliquot of the RNA used for the CDC H5 subtyping assay was thawed from −80°C just before use for ddPCR.

## RESULTS

Using the inclusivity testing protocol, we tested five commonly used POC molecular tests from three manufacturers and 10 LFAs (for POC and/or OTC use), all with EUA or 510k clearance and commercially available, for their ability to detect the 2024 HPAI H5N1 strain as influenza A. As seen in Fig. 1 and Tables S1 and S2, all of the POC molecular assays (Cepheid Xpert Xpress CoV-2/Flu/RSV *plus* test, Roche cobas Influenza A/B & RSV, and Roche cobas SARS-CoV-2 & Influenza A/B for use with the Liat System and the Abbott ID NOW Influenza A & B 2 and Abbott ID NOW COVID-19 2.0 with Influenza A & B 2 sequential workflow) detected heat-inactivated 2024 HPAI H5N1, with the lowest concentration detected ranging from 1.55 to 7.75 TCID_50_ per swab. Currently, the CDC Influenza Virus Real-Time RT-PCR Panel is the only FDA 510k-cleared molecular test for subtyping influenza A(H5) virus and thus provided a useful reference point for the POC molecular assay assessment (Fig. 1; Tables S1 and S2). Notably, a positive result for H5 on the CDC subtyping assay requires positive results for three targets, including an influenza A target and two H5-specific targets (Tables S1 and S2). The Ct values for the CDC assay performed with live versus heat-inactivated virus were within 0.5 Ct for all three targets and dilutions, indicating that heat inactivation of virus did not impact detection (data not shown). Data for sample concentrations expressed in genomic equivalents (GE)/mL, as generated by ddPCR, are provided in Tables S1 and S2.

All 10 LFAs tested detected live 2024 HPAI H5N1, with the lowest concentration detected ranging from 78 TCID_50_ per swab to 1,550 TCID_50_ per swab (Fig. 1; Table S3). Additionally, two LFAs capable of detecting specifically influenza A(H5) were evaluated. The lowest concentration of live 2024 HPAI H5N1 detected by the Healgen FluA (H5) Ag Rapid Test Cassette (Dropper&Swab) RUO assay was 775 TCID_50_/swab (15,000 TCID_50_/mL) (Table S3). This H5 RUO test did not show cross-reactivity to either H1N1 or H3N2 strains at the highest possible concentrations (data not shown). The Arbor Vita AV Avantage A/H5N1 Flu Test (utilizing a revised workflow and IFU relative to the 2009 510k-cleared version, as provided by the manufacturer) did not detect the most concentrated sample of the live 2024 HPAI H5N1 (15,500 TCID_50_/swab, added to 3 mL M6 transport media). When evaluated with an alternative workflow utilizing live viral stock/PNSM mixed 1:1 with M6 (supplemental methods), the AV test detected an amount of virus equivalent to 232,500 TCID_50_ in 3 mL of transport media (77,500 TCID_50_/mL).

As shown in Fig. 1, the influenza A LFAs varied in their sensitivity for detection of the live 2024 HPAI H5N1 strain. The Healgen Rapid Check COVID-19/Flu A&B Antigen Test 510k assay was the most sensitive and detected down to 78 TCID_50_/swab in inclusivity testing, while the least sensitive LFA (LFA B) detected down to 1,550 TCID_50_/swab. Table 1 compares the relative sensitivities of the LFAs for detection of the 2024 HPAI H5N1 strain using a “relative ratio,” with the sensitivity (lowest concentration detected in inclusivity testing) of the Healgen Rapid Check COVID-19/Flu A&B antigen test (the LFA with *de novo* authorization) as the reference (relative ratio = 1). Relative ratios for the sensitivity of detection of 2024 HPAI H5N1 ranged from 2 to 20 (Table 1). For comparison, we also evaluated the relative sensitivity of the LFAs for detection of a 2009 H5N1 strain (Mallard/Wisconsin/2576/2009) (Table 1, column H5N1 [2009]). Inclusivity data for the 2009 H5N1 strain were publicly available for seven LFAs and newly generated at Emory for LFA B. Relative ratios for detection of the 2009 H5N1 strain similarly ranged between 0.2 (Watmind) and 10 (LFA B). The CDC assay again provided a useful reference for determining Ct values in samples used for evaluation of the LFAs (Table S3). Table S3 also indicates ddPCR results (in genomic equivalents/mL) for each sample tested.

**TABLE 1:** Relative sensitivity of LFAs for detection of 2024 HPAI H5N1 and 2009 H5N1 strains^*a*^

LFA name	2024 HPAI H5N1		2009 H5N1	
	A/Bovine/Ohio/B24OSU439/2024 (TCID_50_/mL)	Relative ratio	A/Mallard/Wisconsin/2576/2009 (CEID_50_/mL)	Relative ratio
Healgen Rapid Check COVID-19/Flu A&B Antigen Test (510k^#^)	1,550	1.00	800,000	1.00
Quidel Sofia 2 Flu + SARS Antigen FIA (EUA)	3,100	2.00	Data not available	
ACON Laboratories Flowflex Plus COVID-19 and Flu A/B Home Test (510k^#^)	3,100	2.00	Data not available	
Sekisui Diagnostics OSOM Flu SARS CoV 2 Combo Test (EUA^#^)	3,100	2.00	1,600,000	2.00
Watmind USA SpeedySwab Rapid COVID-19 + Flu A&B Antigen Self-Test (EUA^#^)	3,100	2.00	160,000	0.20
Wondfo USA WELLlife COVID-19/Influenza A&B Home Test (510k^#^)	3,100	2.00	400,000	0.50
CorDx Tyfast Flu A/B & COVID-19 Multiplex Rapid Test (EUA^#^)	7,750	5.00	800,000	1.00
OHC COVID-19/Flu Antigen Test Pro (510k^#^)	7,750	5.00	4,000,000	5.00
LFA (A)	15,500	10.00	1,600,000	2.00
LFA (B)	31,000	20.00	8,000,000*	10.00

^*a*^ All data for A/ Bovine/Ohio/B24OSU439/2024 (HPAI) H5N1 (2024) were generated at Emory. Only data for LFA (B) for the A/Mallard/Wisconsin/2576/2009 H5N1 (2009) strain (*) were generated by Emory, while other data for that strain were publicly available in assay IFUs. # indicates tests cleared/approved or authorized by the FDA for home use. LFA (A) and LFA (B) are anonymized and, therefore, 510k/EUA status and authorized use settings cannot be disclosed. LFA, lateral flow assay; HPAI, highly pathogenic avian influenza; IFU, instructions for use; EUA, emergency use authorization; TCID_50_/mL, tissue culture infectious dose (measure of virus concentration that will infect 50% of cells in tissue culture); CEID_50_/mL, chicken embryo infectious dose (measure of virus concentration that will infect 50% of chicken embryos in culture). Data for H5-specific LFAs were not included.

To understand whether the observed variations in sensitivity for H5N1 detection were unique to H5N1, we examined the relative sensitivity of the influenza A LFAs for the seasonal flu strains (H1N1 and H3N2) that they were designed to detect (Table 2). Importantly, the range and distribution of relative ratios for detection of live H1N1 and H3N2 were very similar to the relative ratios for detection of the live 2024 HPAI and 2009 H5N1 strains. This indicates that while the LFAs do have some variation in their ability to detect H5N1, they demonstrated a very similar and stable variation in their ability to detect the seasonal influenza A strains for which they were designed.

**TABLE 2:** Relative sensitivity of LFAs for the detection of seasonal influenza strains (H1N1 and H3N2)^*a*^

LFA name	H1N1				H3N2			
	A/California/ 04/2009 (TCID_50_/mL)	Relative ratio	A/Ohio/ 09/2015 (CEID_50_/mL)	Relative ratio	A/Tasmania/ 503/2020 (FFU/mL)	Relative ratio	A/Indiana/ 08/2011 (TCID_50_/mL)	Relative ratio
Healgen Rapid Check COVID-19/Flu A&B Antigen Test (510k^#^)	2,800*	1.00	700,000	1.00	65,000*	1.00	810	1.00
Quidel Sofia 2 Flu + SARS Antigen FIA (EUA)	Data not available				Data not available			
ACON Laboratories Flowflex Plus COVID-19 and Flu A/B Home Test (510k^#^)	Data not available		1,750,000**	2.50	162,500**	2.50	810**	1.00
Sekisui Diagnostics OSOM Flu SARS CoV 2 Combo Test (EUA^#^)	3,500	1.25	3,500,000	5.00	330,000	5.08	4,100	5.06
Watmind USA SpeedySwab Rapid COVID19 + Flu A&B Ant-igen Self-Test (EUA^#^)	Data not available		700,000	1.00	33,000	0.51	200	0.25
Wondfo USA WELLlife COVID-19/Influenza A&B Home Test (510k^#^)	2,800	1.00	700,000	1.00	130,000	2.00	810	1.00
CorDx Tyfast Flu A/B & COVID-19 Multiplex Rapid Test (EUA^#^)	Data not available		7,000,000	10.00	130,000	2.00	2,000	2.47
OHC COVID-19/Flu Antigen Test Pro (510k^#^)	2,800	1.00	1,400,000	2.00	130,000	2.00	810	1.00
LFA (A)	14,000	5.00	1,400,000	2.00	130,000	2.00	810	1.00
LFA (B)	140,000**	50.00	14,000,000**	20.00	650,000**	10.00	40,500**	50.00

^*a*^ Data indicated with (*) were obtained from IFUs and confirmed by testing performed at Emory. Data indicated with (**) were generated at Emory, while other data for that strain were obtained from publically available assay IFUs. # indicates tests cleared/approved or authorized by the FDA for home use. LFA (A) and LFA (B) are anonymized; therefore, 510k/EUA status and authorized use settings cannot be disclosed. LFA, lateral flow assay; HPAI, highly pathogenic avian influenza; IFU, instructions for use; EUA, emergency use authorization; TCID_50_/mL, tissue culture infectious dose (measure of virus concentration that will infect 50% of cells in tissue culture); CEID50/mL, chicken embryo infectious dose (measure of virus concentration that will infect 50% of chicken embryos); FFU/mL, focus forming units per milliliter. H5 specific LFAs were not tested.

To assess the impact of gamma irradiation on viral antigen detection and facilitate evaluation by laboratories lacking BSL3 facilities, a subset of LFAs (those with 510k clearance, detecting nucleoprotein [NP], and the Healgen H5-specific RUO LFA) were tested with gamma-inactivated 2024 HPAI H5N1 available from BEI (see Materials and Methods), which was generated from the same virus stock used for live virus testing in Fig. 1. Results indicated that gamma inactivation does not significantly affect antigenicity of the nucleoprotein (NP) target protein, with the lowest concentration detected by three influenza A LFAs ranging from 39 to 388 TCID_50_ per swab (Table S4). The hemagglutinin (HA) target (for the H5-specific RUO LFA) appeared to be slightly more affected by gamma irradiation, with the lowest concentration detected increasing fivefold (from 775 to 3,875 TCID_50_/swab). To assess the impact of gamma inactivation on molecular detection, the CDC assay was performed with live virus and GIV for all dilutions tested with LFAs. The CDC assay was positive for all dilutions used for LFA testing. Cts of live versus gamma-inactivated virus were within 0.1 to 4 Ct for all four targets (Table S3 versus S4).

Finally, live 2024 HPAI H5N1 D1.1, a genotype circulating in poultry and wild birds and more recently detected in dairy cattle in Nevada and Arizona (15), was used to test the same four LFAs that were tested with GIV 2024 HPAI H5N1. All four tests were inclusive for 2024 HPAI H5N1 D1.1 (Table S5).

## DISCUSSION

This study primarily evaluated the ability of commercially available rapid antigen (lateral flow) and molecular influenza A assays for use in POC and/or OTC settings to detect the 2024 HPAI H5N1 strain circulating in dairy cattle in the U.S. All tests selected for the study are “combo” tests able to detect not only influenza A but also SARS-CoV-2, influenza B, and in some cases, RSV. Tests were compared to the CDC influenza A/H5 assay (implemented as a research-use only [RUO] application), and the use of consistent reagents and protocols enabled a direct head-to-head comparison of assay performances.

Using the standard inclusivity testing protocol, our data demonstrated that all of the commercially available influenza A LFAs tested were able to detect the 2024 HPAI H5N1 strain (genotype B3.13) in contrived samples within a 20-fold range of sensitivity. Additional testing of four of the LFAs (510k and Healgen H5-specific RUO LFA) confirmed that they were also able to detect a 2024 HPAI H5N1 D1.1 (a genotype circulating in poultry and wild birds). Notably, the range and relative differences in analytical sensitivity for the 2024 HPAI H5N1 strain were observed to be similar for detection of a 2009 H5N1 strain and seasonal influenza A strains (H1N1 and H3N2), indicating reproducible intrinsic differences in the sensitivity of the tests evaluated. While this range of analytical sensitivity is likely to translate into a range of clinical sensitivity, it is important to note that all the influenza A LFAs evaluated in this study have already been cleared or authorized for the detection of seasonal influenza with support from large prospective clinical trials. Notably, we also observed that an RUO LFA (Healgen) designed to be specific for H5 detection and optimized for detection of a 2024 H5N1 HA sequence was able to detect both the 2024 HPAI H5N1 B3.13 and 2024 HPAI H5N1 D1.1 strains with analytical sensitivity similar to the commercially available influenza A LFAs, raising the possibility of the future use of such a test (once authorized/cleared) either for primary testing or for influenza H5 subtyping following influenza A detection by LFA.

Noting that test manufacturers would benefit from the ability to use inactivated viral stock reagents for test development, we also evaluated the ability of selected tests to detect the BEI gamma-irradiated virus (GIV) derived from the live 2024 HPAI H5N1 strain. As expected, there was no apparent impact of gamma irradiation on molecular detection with the CDC assay. While the GIV 2024 HPAI H5N1 strain was detected by the three 510k-cleared influenza A LFAs (which detect a NP target) with sensitivity equivalent to the live strain, we did note that the Healgen H5-specific RUO LFA detected the GIV strain with fivefold less sensitivity than the live strain, suggesting that developers of tests detecting the HA target should test their assays against live H5 viruses. We acknowledge that the use of frozen contrived specimens for testing may have slightly impacted sensitivity measurements but made the head-to-head comparison of test performances possible.

All of the POC molecular assays evaluated were shown to be inclusive for the 2024 HPAI H5N1 strain, with lowest concentrations detected within a fivefold range.

These analytical data are key to preparedness, enabling rapid response in the event of increased human-to-human transmission of H5N1. To date, almost all of the U.S. H5N1 cases have been limited to individuals directly exposed to infected animals, and clear evidence of human-to-human transmission has not been observed. The commercial assays evaluated in this study are cleared/authorized for anterior nares (AN) swab specimens or, in the case of the POC molecular assays, AN and nasopharyngeal (NP) swab specimens. The clinical performance of these assays in patients with H5N1 infection, many of whom have presented with conjunctivitis (16–18), is currently unknown due to a lack of H5N1 clinical samples for evaluation. To date, concentrations of the virus in PCR-positive clinical samples have been relatively low (mean Ct value of 31.0) (16). However, if there were to be evolution of this virus such that increased human-to-human transmission was observed, the sample types optimal for clinical testing (and viral concentrations in those samples) would need to be defined.

Our inclusivity testing data provide support for our national and global efforts to prepare for H5N1 influenza and demonstrate the utility of expeditious head-to-head evaluation of available tests using consistent reagents and protocols. In the event of an emergence of an evolved strain of H5N1 with higher rates of human-to-human transmission, it would be prudent to evaluate the performance of these assays using the new strain and additional types of clinical samples (19).

### Summary

Recent human infections with H5N1 influenza necessitate diagnostic preparedness. Reassuringly, commercially available rapid antigen and point-of-care molecular influenza tests detected the 2024 U.S. bovine H5N1 strain in contrived specimens. Clinical performance should be evaluated if human-to-human transmission is observed.

## SUPPLEMENTAL MATERIAL

Supplemental materials jcm.00548-25-s0001.docx Supplemental methods and Tables S1 to S5.

ASM does not own the copyrights to Supplemental Material that may be linked to, or accessed through, an article. The authors have granted ASM a non-exclusive, world-wide license to publish the Supplemental Material files. Please contact the corresponding author directly for reuse.
